# Tertiary Syphilitic Gumma Mimicking Testicular Neoplasms

**DOI:** 10.7759/cureus.37392

**Published:** 2023-04-10

**Authors:** Markus Angerer, Felix Lübbersmeyer, Raphael Gübitz, Christian Wülfing, Klaus-Peter Dieckmann

**Affiliations:** 1 Department of Urology, Asklepios Klinik Altona, Hamburg, DEU; 2 Department of Urology, Medical School Hamburg, Hamburg, DEU; 3 Department of Radiology, Asklepios Klinik Altona, Hamburg, DEU

**Keywords:** gumma, treponema pallidum, orchidectomy, testis-sparing management, bilateral testicular masses, germ-cell tumor, tertiary syphilis

## Abstract

Palpable testicular masses in men aged 20 to 50 years usually represent testicular germ cell tumors. Diagnostic work-up involves ultrasound examination as well as serum tumor markers alpha fetoprotein, beta-human chorionic gonadotropin and lactate dehydrogenase, and particularly the novel marker M371. Orchidectomy is mandatory for germ cell tumors. We report the rare case of testicular involvement by tertiary syphilis mimicking testicular neoplasms with testis-sparing management.

A 46-year-old Caucasian male presented with a painless firm mass in the right testicle and multiple cutaneous plaques at the skin of the scrotum, penis and right forearm. Testicular serum tumor markers were negative. Syphilis Rapid Plasma Reagin test and Treponema pallidum immunoglobulin antibodies tests were positive. Radiological examination revealed bilateral testicular lesions as well as bipulmonal pleural-based opacities. Conservative management was attempted and treatment with ceftriaxone (2 g/day) intravenously for 14 days was administered. The testicular findings improved rapidly and significantly during antibiotic treatment. Radiological follow-up examinations after two weeks and two months showed further regression of the testicular and pulmonary lesions.

This case represents an extremely rare testicular manifestation of tertiary syphilis. Due to rising syphilis incidence in Europe, tertiary syphilis with formation of gumma should be a differential diagnosis of testicular tumor. Thus, syphilis-specific treatment is safe and orchidectomy can be avoided.

## Introduction

Painless palpable testicular masses in men aged 20 to 50 years usually represent testicular germ cell tumors. Diagnostic work-up involves ultrasound examination showing irregular shaped hypoechogenic areas with positive findings of color-coded Doppler sonography within the testis. Further support comes from elevations of serum tumor markers alpha fetoprotein (AFP), beta-human chorionic gonadotropin (β-hCG) and lactate dehydrogenase (LDH). The diagnosis is finally established by surgical exploration with the histological confirmation of a germ cell tumor. In these cases radical orchidectomy is mandatory. Preoperative differential diagnoses involve epididymitis, hydrocele testis, scrotal hernia and rarely swellings secondary to vascular disorders, trauma, or chronic granulomatous/inflammatory diseases [1]. 

Herein, we report the rare case of testicular involvement by tertiary syphilis mimicking testicular neoplasms.

## Case presentation

A 46-year-old healthy Caucasian man presented with a painless firm mass in the right testicle existing since three weeks. At the same time, the patient developed multiple cutaneous plaques at the skin of the scrotum, penis and right forearm. The patient frankly reported to be homosexual. Medical history included a recognized syphilis infection about 20 years ago, which had been treated with antibiotics (product name patient unknown). There was necrotizing fasciitis of the left thigh two years ago managed surgically. The patient also reported a penicillin allergy. Antibiotic treatment with ciprofloxacin for the cutaneous lesions had been initiated by the family physician some days earlier. HIV testing had repeatedly been negative with the last test performed a few months ago.

**Figure 1:**
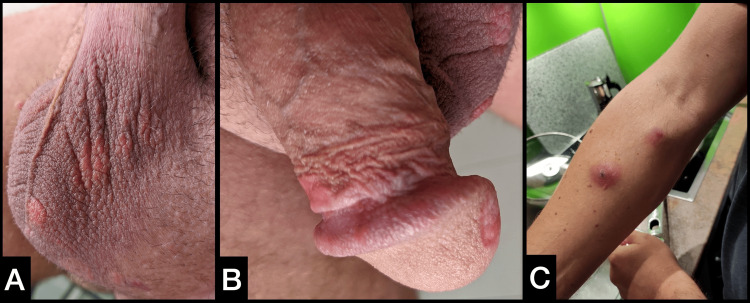
Cutaneous lesions Multiple cutaneous lesions of the scrotum (A), penis (B) and right forearm (C)

**Figure 2:**
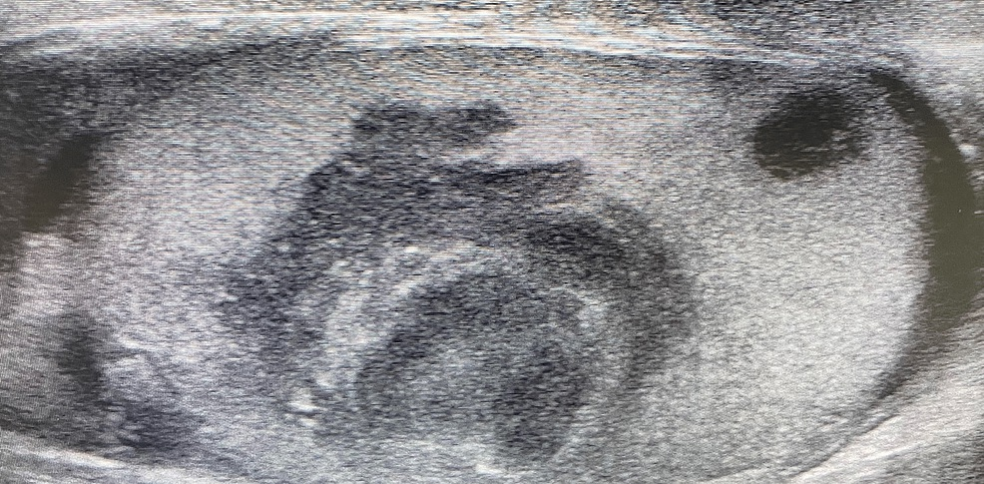
Scrotal sonography at hospitalization Right testis with hypoechogenic mass (up to 30 mm in diameter) in the middle third and smaller hypoechogenic lesion at the lower pole

Upon physical examination, the patient appeared in a good general condition. There were several small penile and scrotal cutaneous plaques, and two cutaneous lesions on the right forearm sized about 1 cm each (Figure 1A-1C). Inguinal, subclavicular and cervical lymph nodes were not palpable enlarged on both sides. Scrotal sonography disclosed multiple rounded and well-demarcated hypoechogenic lesions up to 10 mm in size in both testes. The largest mass was approximately 30 mm in diameter with only weak signals in color-coded Doppler sonography in the right testis (Figure 2).

**Figure 3:**
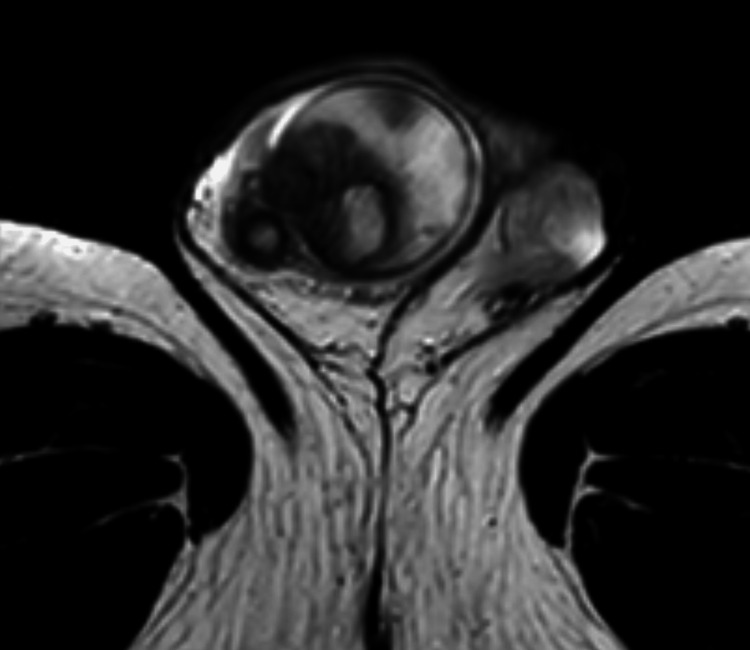
T2-weighted MRI of the pelvis at hospitalization Transversal view showing right testicular lesions with central hyperintensity and peripheral hypointensity. The right epididymis shows tubular dilatation with central hyperintensity and peripheral hypointensity

**Figure 4:**
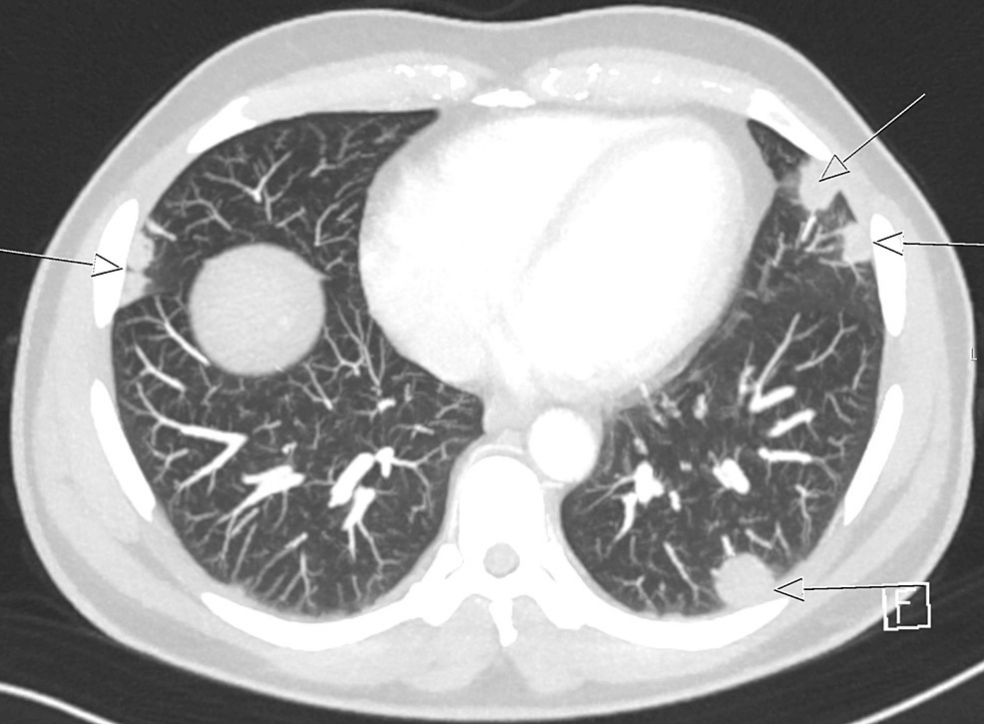
CT scan of the chest at hospitalization Transversal image showing labeled bipulmonal pleural-based opacities with the largest lesion up to 21 mm in diameter

Upon MRI of the pelvis, both testes showed multiple lesions, which were hyperintense in the center and hypointense in the periphery on T2-weighted imaging (Figure 3). Administration of contrast agent demonstrated strong peripheral vascularity and central hypointensity of the lesions. The findings were considered as centrally necrotic lesions with surrounding inflammatory changes. As the primary differential diagnosis was testicular malignancy, CT scan of chest and abdomen was performed. It revealed bipulmonal pleural-based opacities up to 2.1 cm in diameter with heterogeneous enhancement or central hypodensity (Figure 4). In view of the entire spectrum of clinical findings of the patient, the CT images were interpreted as necrotic masses caused by infection.

Laboratory work-up revealed serum tumor markers AFP, β-hCG and LDH as well as white blood cells to be within normal limits. C-reactive protein (CRP) was slightly increased. The treponemal screening test gave a positive result. Rapid Plasma Reagin test (1:64) as well as Treponema pallidum (TP) immunoglobulin (Ig) M antibodies (1:160) and TP IgG antibodies (1:25600) showed elevated titers (Table 1). Tests for HIV, Chlamydia trachomatis and Neisseria gonorrhoeae gave negative results. Urine microscopy was negative for Mycobacterium tuberculosis. Urine culture remained sterile.

**Table 1:** Laboratory work-up AFP = alpha fetoprotein; β-HCG = beta-human chorionic gonadotropin; CRP = C-reactive protein; WBC = white blood cells; IFT = immunofluorescence test;  LDH = lactate dehydrogenase; neg = negative; pos = positive; quant = quantitative; RPR = Rapid Plasma Reagin test; semiquant = semiquantitative; TP = Treponema pallidum

Laboratory parameters	Hospitalization	Discharge	Two week follow up	Two month follow up	Reference
CRP	9,3 mg/L	1,4 mg/L	8,0 mg/L	1,2 mg/L	< 5,0 mg/L
WBC	5,3/nl	4,1/nl	5,7/nl	5,8/nl	3,5 - 9,8/nl
ß-HCG	< 1,20 U/L	-	-	< 1,20 U/L	< 2,0 U/L
AFP	3,2 µg/L	-	-	4,5 µg/L	< 8,0 µg/L
LDH	231 U/L	-	-	213 U/L	< 250 U/L
Primary screening test	pos.	pos.	pos.	pos.	neg.
RPR	1:64	> 1:2	> 1:2	> 1:2	nonreactive
RPR semiquant	1:64	1:64	1:32	1:32	nonreactive
TP IgG IFT, serum	-	-	-	> 1:1600	neg.
TP IgG IFT, serum, quant.	1:25600	1:25600	1:25600	1:12800	neg.
TP IgM IFT, serum	-	-	-	> 1:40	neg.
TP IgM IFT, serum, quant.	1:160	1:640	1:320	-	neg.

Ultimately, laboratory work-up disclosed an active syphilis infection and the final diagnosis of tertiary syphilis with testicular, pulmonary and skin involvement was made. Hence, conservative management was attempted and treatment with ceftriaxone (2 g/day) intravenously for 14 days was administered.

The testicular findings improved rapidly and significantly during antibiotic treatment. After two weeks of treatment, the cutaneous manifestations on the penis, scrotum and forearm had almost disappeared, but the testicular lesions were almost unchanged at that time. A three-week regimen of cotrimoxazole was applied after completion of intravenous ceftriaxone treatment.

**Figure 5:**
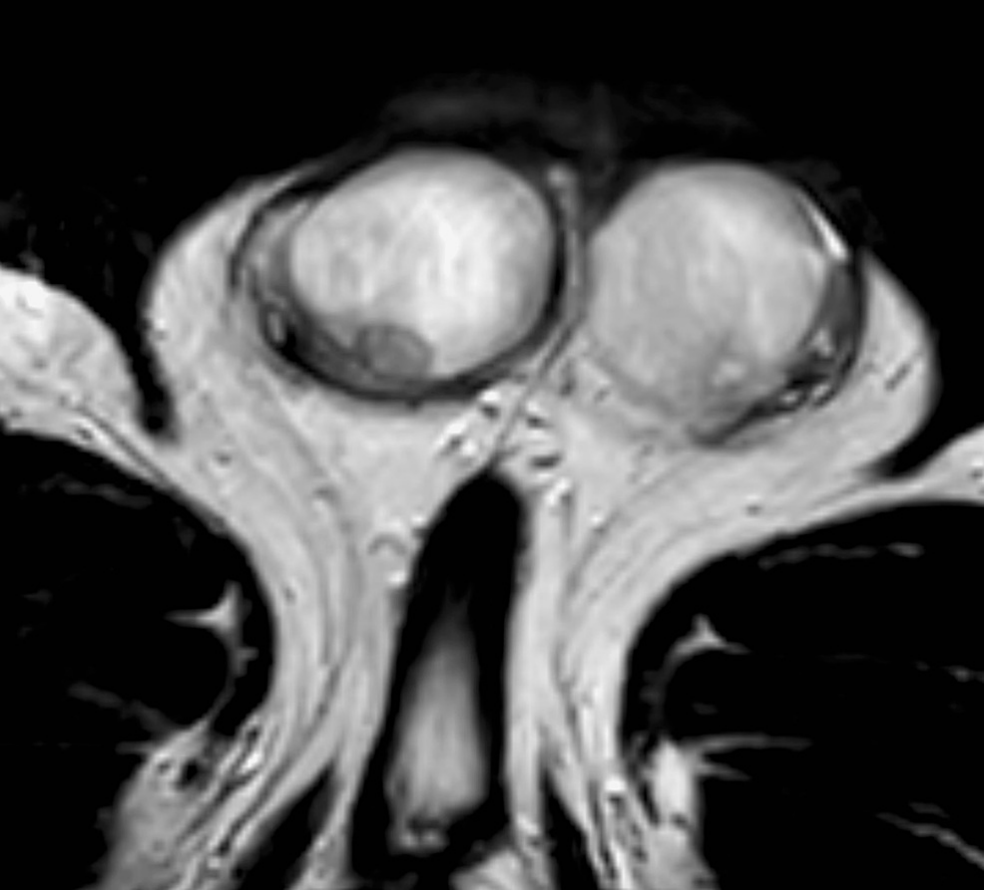
T2-weighted MRI of the pelvis at two-month follow up Transversal image showing significant reduction of both number and size of testicular lesions

**Figure 6:**
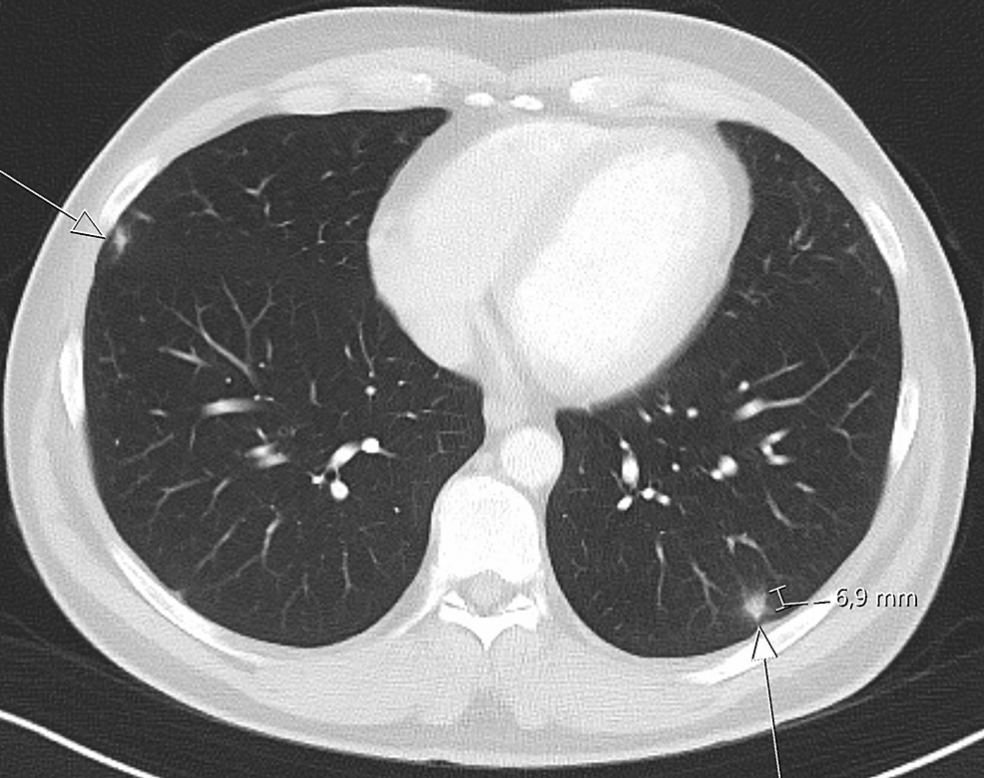
CT scan of the chest at two-month follow up Transversal image showing significant labeled regression of the lesions with the largest lesion up to 6.9 mm in diameter

Follow-up examinations after two weeks and two months showed decreased swelling of the testis with persisting firmness, clinically. Ultrasound and pelvic MRI disclosed significant reduction in both number and size of testicular lesions (Figure 5). Follow-up chest CT after two months revealed marked regression of the round-shaped lesions of the lung (Figure 6). We assume that the disappeared cutaneous and pulmonary lesions were syphilitic gummas.

## Discussion

Syphilis is an infectious disease caused by the spirochaete Treponema pallidum [14]. The dominant way of transmission is sexual (vaginal, anal, oral), but infection may also be acquired transplacentally or through contact with contagious material or blood products [14]. Recent epidemiological studies demonstrated rising incidence of syphilis in Europe. Almost three out of four infected people are men who have sex with men. Syphilis rates are nine times higher in men than in women. In 2019, the registration rate for syphilis was 7.4 cases per 100,000 population per year in Europe. The highest incidence is encountered in 25 - 34-year-old men [15].

Typically, untreated syphilis progresses in four stages. The primary stage is characterized by a usually painless sore, called chancre, on genitals, rectum, tongue or lips and regional lymphadenopathy [14]. The chancre develops with a mean time of three weeks after infection and disappears without treatment after four to six weeks [14]. Secondary syphilis (four to 10 weeks later) causes a generalized nontender lymphadenopathy and macular rash that can cover the entire body [14]. Other symptoms can include fever, weight loss, fatigue, mucosal lesions, alopecia and condylomata lata [14]. During early and late latent syphilis patients are often asymptomatic, but remain positive in serological tests [14]. In one-third of all untreated cases, the tertiary syphilis stage is reached after 15 to 30 years [14]. Based on a granulomatous tissue reaction patients develop cutaneous nodules and syphilitic gummas that can occur in every organ [14]. Tertiary syphilis can include cardiovascular (e.g. aortitis, myocardial ischemia) and neurological manifestations (e.g. personality changes, meningoencephalitis, pareses) [14]. Quaternary syphilis, also named neurosyphilis, is the manifestation of the central nervous system including meningitis or vasculitis causing hemiparesis, paraplegia, aphasia or seizure. Neurosyphilis can be found in early and late stages of syphilis [16]. Very rarely, syphilis can affect the testis presenting with clinical and imaging features that are hardly discernible from malignant testicular tumors.

The final diagnosis of syphilis can be made by direct detection methods for TP [17]. The European guideline on the management of syphilis recommends a primary screening test followed by a confirmatory test if screening shows a positive result [17]. Suitable screening tests are treponemal tests such as TP haemagglutination test (TPHA), TP particle agglutination test (TPPA) and fluorescent treponemal antibody absorption test (FTA-abs test) [15]. These tests are lifelong positive in most patients [17], and accordingly, the FTA-abs test was positive in the present patient, too. These tests are unable to detect active TP infection or successful treatment [18]. For confirmation of active syphilis, the Venereal Disease Research Laboratory test (VDRL) and or Rapid Plasma Reagin test (RPR) are recommended by guidelines [17]. Patients with active infection show a reactive RPR test result (cut off 1:1). In our patient, RPR titer was clearly elevated at 1:64.

Standard treatment for early and late stages of syphilis is benzathine penicillin G (BPG): in primary, secondary and early latent syphilis, BPG 2.4 million units intramuscularly on day one is recommended [17]. In late latent, cardiovascular and gummatous syphilis, BPG 2.4 million units intramuscularly on days one, eight and 15 is recommended [17]. Intravenous administration is preferred for neurosyphilis [17]. Penicillin allergy or bleeding disorders require alternative therapy with ceftriaxone (in early stages 1 g/day intravenously for 10 days) or doxycycline [17]. In our patient, ceftriaxone was administered because of penicillin allergy.

Tertiary syphilis commonly involves skin, cardiovascular system and nervous system [14]. Testicular involvement in tertiary syphilis is extremely rare with only 12 cases reported in the English literature since 1947 (Table 2). Testicular manifestation of syphilis can present as uni-, bilateral, single or multiple lesions, as well as diffuse infiltration of the testis [5,11]. The testicular swelling is often painless, smooth and firm [5]. The adjacent epididymis can be affected [8,9]. In the present case, there was a bilateral testicular and multiorgan (lungs) manifestation.

**Table 2:** Overview of History Previously published cases of tertiary syphilis with testicular manifestation in English language. BPG = benzathine penicillin G; d = days; HIV = human immunodeficiency virus; i.m. = intramuscularly; i.v. = intravenously; N/S = not specified; neg = negative; pos = positive; yr = year

Author (yr of publication)	Patient age (in yr)	Sexuality	HIV status	Testicular findings	Other manifestations	Treatment
London (1947) [2]	33	N/S	N/S	Bilateral testicular hardening and swelling initially painful	Fever and general malaise	Mapharsen i.v., bismuth subsalicylate i.m. left orchidectomy
Lees (1957) [3]	27	Heterosexual	N/S	Bilateral painless testicular swelling, scrotal ulcer	None	Iodide, neoarsphenamine, bismuth
Persaud et al. (1977) [4]	59	N/S	N/S	Painful swelling and hardening of the right testis and spermatic cord	None	Right orchidectomy + long-acting penicillin for 10 d
Al-Egaily (1977) [5]	37	Heterosexual	N/S	Bilatera enlarged, firm and slightly painful testes	Asymptomatic neurosyphilis, penile ulcers	Procaine penicillin i.m. for 17 d
Dao et al (1980) [6]	50	N/S	N/S	Bilateral enlargement and painless hardening	Neurosyphilis, aortic fibrosis	None
Samuels et al (1991) [7]	35	Heterosexual	Neg.	Unremarkable	Neurosyphilis	Left orchidectomy + penicillin i.v. for 14 d, BPG i.m. in 4 weekly doses
De Silva et al. (2010) [8]	32	Heterosexual	Neg.	Bilateral swelling, left epididymis thickened, left hydrocele	None	BPG i.m. in 2 weekly doses, right orchidectomy + procaine penicillin i.m. for 17 d
Mackenzie et al. (2011) [9]	47	Heterosexual	N/S	Painful right testicular swelling, right epididymis thickened	None	Ciprofloxacin for 14 d, right orchidectomy + BPG i.m. in 3 weekly doses
Liang et al. (2013) [10]	37	Homosexual	Pos.	Painless left testicular enlargement and hardening	Left nephropathy	Left orchidectomy + doxycycline for 30 d
Chu et al. (2016) [11]	33	N/S	Pos.	Right testicular swelling and hardening	None	Right orchidectomy
Agrawal et al.(2019) [12]	40	N/S	Pos.	Painful and swollen left hemiscrotum with abscess	None	Left orchidectomy + ceftriaxone, metronidazole, BPG i.m.
Nepal et al. (2021) [13]	63	N/S	Neg.	Bilateral painless, firm and smooth testes	Bilateral pleural effusion, vasculitis, osteomyelitis, dermatopanniculitis	Left orchidectomy + ampicillin for 14d

Testicular manifestation of tertiary syphilis is diagnosed by clinical findings and positive syphilis serology [5]. Improvement under antibiotic treatment verifies the diagnosis [5]. Important differential diagnoses involve other infectious diseases e.g. tuberculous epididymitis, gonococcal epididymitis, non-specific epididymitis and mumps orchitis [5].

However, the differentiation of testicular gummata from malignant testicular tumor remains the greatest challenge [5]. In our case, ultrasound demonstrated bilateral hypoechogenic lesions with weak signals in color-coded Doppler sonography. In addition, MRI (T2-weighted imaging) showed central hyperintense and periphery hypointense lesions. They demonstrated strong peripheral vascularity and central hypointensity after administration of contrast agent. The bilateral multifocal involvement in conjunction with imaging features suggested an inflammatory disease rather than malignant neoplasm. This is the first syphilitic gumma with MRI imaging of a patient with no evidence of neurosyphilis and associated symptoms.

Radical orchidectomy of the affected testis was performed in most of the cases reported to date with suspected malignancy being the reason for surgery. However, as demonstrated in the present case, orchidectomy with histologic examination is not required for establishing the diagnosis of tertiary testicular syphilis [5], and this rule particularly applies to patients with positive syphilis history and high probability for testicular involvement of syphilis. The diagnosis can be secured by combination of medical history, and laboratory findings with absence of serum tumor markers including absence of microRNA-371 as well as positive findings of the treponemal screening test and additionally elevated titers in the RPR. Moreover, diagnosis can be confirmed by response to antibiotic therapy, which will become evident after a few weeks only (“diagnosis ex iuvantibus”). Even given the worst case scenario, that an early stage malignant germ cell tumor is mimicking testicular syphilis, a delay of surgery of three or four weeks does usually not involve detrimental effects on the chance of cure [19].

The present case demonstrates that testis-sparing management is in fact feasible in cases with testicular syphilitic lesions. Diagnosis is to be based on the criteria outlined by Al-Egaily [5]. Antibiotic treatment can cause significant remission of space-occupying intratesticular lesions as seen in the present case who is well and without symptoms months after initiation of conservative treatment. The repeated imaging has shown a significant reduction in size and number of testicular and pulmonary lesions after two-month follow-up as shown in Figures 5, 6. Therefore, conservative management will be continued. 

## Conclusions

This case represents an extremely rare testicular manifestation of tertiary syphilis. Due to rising syphilis incidence in Europe, tertiary syphilis with formation of gumma should be a differential diagnosis of testicular tumor, particularly in risk groups of sexually transmitted infections. The diagnosis is established by serological screening test e.g. TPHA and with documentation of IgM and IgG antibodies with the RPR. Conservative syphilis treatment with antibiotics is safe. Therefore (radical) orchidectomy can usually be avoided.
